# Floral scent emission of *Epiphyllum oxypetalum*: discovery of its cytosol-localized geraniol biosynthesis

**DOI:** 10.1093/hr/uhaf039

**Published:** 2025-02-11

**Authors:** Yiyang Zhang, Yuhan Zhang, Andong Zhang, Qiurui Tian, Bin Yang, Likun Wei, Wei Wu, Ting Zhu, Zhiwei Zhou, Jiaqi Wang, Zhibin Liu, Wei Tang, Haijun Xiao, Mingchun Liu, Tao Li, Qun Sun

**Affiliations:** Key Laboratory of Bioresources and Eco-environment of Ministry of Education, College of Life Sciences, Sichuan University, No.29 Wangjiang Road, Chengdu, Sichuan, China; Peking University-Tsinghua University-National Institute of Biological Sciences Joint Graduate Program, Academy for Advanced Interdisciplinary Studies, Peking University, No.5 Yiheyuan Road, Beijing, China; Key Laboratory of Bioresources and Eco-environment of Ministry of Education, College of Life Sciences, Sichuan University, No.29 Wangjiang Road, Chengdu, Sichuan, China; Key Laboratory of Bioresources and Eco-environment of Ministry of Education, College of Life Sciences, Sichuan University, No.29 Wangjiang Road, Chengdu, Sichuan, China; Key Laboratory of Bioresources and Eco-environment of Ministry of Education, College of Life Sciences, Sichuan University, No.29 Wangjiang Road, Chengdu, Sichuan, China; Key Laboratory of Bioresources and Eco-environment of Ministry of Education, College of Life Sciences, Sichuan University, No.29 Wangjiang Road, Chengdu, Sichuan, China; Key Laboratory of Bioresources and Eco-environment of Ministry of Education, College of Life Sciences, Sichuan University, No.29 Wangjiang Road, Chengdu, Sichuan, China; Key Laboratory of Bioresources and Eco-environment of Ministry of Education, College of Life Sciences, Sichuan University, No.29 Wangjiang Road, Chengdu, Sichuan, China; Key Laboratory of Bioresources and Eco-environment of Ministry of Education, College of Life Sciences, Sichuan University, No.29 Wangjiang Road, Chengdu, Sichuan, China; Key Laboratory of Bioresources and Eco-environment of Ministry of Education, College of Life Sciences, Sichuan University, No.29 Wangjiang Road, Chengdu, Sichuan, China; Key Laboratory of Bioresources and Eco-environment of Ministry of Education, College of Life Sciences, Sichuan University, No.29 Wangjiang Road, Chengdu, Sichuan, China; Key Laboratory of Bioresources and Eco-environment of Ministry of Education, College of Life Sciences, Sichuan University, No.29 Wangjiang Road, Chengdu, Sichuan, China; Sichuan Academy of Botanical Engineering, Sichuan Academy of Agricultural Sciences, No.14 Yongxing Road, Chonglong Town, Zizhong, Sichuan, China; Key Laboratory of Bioresources and Eco-environment of Ministry of Education, College of Life Sciences, Sichuan University, No.29 Wangjiang Road, Chengdu, Sichuan, China; Key Laboratory of Bioresources and Eco-environment of Ministry of Education, College of Life Sciences, Sichuan University, No.29 Wangjiang Road, Chengdu, Sichuan, China; Key Laboratory of Bioresources and Eco-environment of Ministry of Education, College of Life Sciences, Sichuan University, No.29 Wangjiang Road, Chengdu, Sichuan, China; Key Laboratory of Bioresources and Eco-environment of Ministry of Education, College of Life Sciences, Sichuan University, No.29 Wangjiang Road, Chengdu, Sichuan, China

## Abstract

*Epiphyllum oxypetalum*, a renowned ornamental species in Cactaceae, releases attractive fragrance during its infrequent, transient, and nocturnal blooms. However, the floral fragrance composition and biosynthesis remain largely unexplored. Employing volatilomics, transcriptomics, and biochemistry, we systematically characterized the composition, emission dynamics, and biosynthesis of the floral scent of *E. oxypetalum*. The floral scent composition of *E. oxypetalum* was highly dynamic. Starting after 8 p.m. local time, volatile emission increased 200-fold within 6 h. At full bloom, geraniol accounted for 72.54% of the total emission, followed by benzyl alcohol (12.96%) and methyl salicylate (3.75%). These scents predominantly originated from petals and sepals. Transcriptomic analysis and inhibition assays using pathway-specific inhibitors revealed that the mevalonate pathway was the precursor source for geraniol biosynthesis. Functionally characterized cytosol-localized geraniol synthase EoTPSa1 was the key enzyme responsible for geraniol biosynthesis. Together, these findings pinpoint a cytosolic biosynthetic route for the major scent volatile geraniol in *E. oxypetalum*. Our study provides new insights into the emission dynamics and biosynthesis of *E. oxypetalum* floral scents. In particular, we demonstrate a distinctive mevalonate pathway-based geraniol biosynthetic pathway, which may hold potential for the development of novel perfume products.

## Introduction

Angiosperm flowers emit diverse and complex fragrances of volatile organic compounds (VOCs) to attract pollinators and repel florivores [1, 2]. The composition and emission patterns of floral VOCs exhibit considerable spatial and temporal variability [3], creating distinct and characteristic fragrances. Species of Cactaceae, which are often cultivated as ornamental flowers, emit diverse floral scents tailored to their pollinators. Diurnal flowers are often scentless for bee- or bird-pollinated cacti; nocturnal-blooming cacti usually emit a contrasting spectrum of volatiles. The bat-pollinated flowers usually produce sulphur-containing VOCs with a cabbage-like smell, whereas moth-pollinated species emit complex blends of terpenoids and benzenoids during their transient nocturnal flowering, creating an appealing cactus aroma [4]. *Epiphyllum oxypetalum*, a popular ornamental species from Cactaceae, emits a strong, pleasant scent that lasts for only a few hours during nocturnal blooming. It begins with a mild phenolic odour, transitioning to a pronounced fragrance. Previous studies have identified several monoterpenes from moth-pollinating cacti, some of which have been widely used in fragrance industries because of their fruity aroma [5]. However, these sporadic reports often omit the dynamic nature and biosynthetic origin of cactus scents, hindering a comprehensive understanding of this highly specialized taxon.

Terpenoid volatiles contribute greatly to the diversity of floral scents [6]. Despite their vast structural diversity, all terpenoid volatiles originate from common five carbon precursors, isopentenyl diphosphate (IPP), and dimethylallyl diphosphate (DMAPP) [7]. Plants employ two distinct and spatially separate pathways to synthesize IPP and DMAPP. The mevalonate (MVA) pathway, which is located in the cytosol and peroxisome, synthesizes these C_5_ building blocks from acetyl-CoA, whereas the methylerythritol phosphate (MEP) pathway, which is located in the plastids, generates these building blocks from pyruvic acid and glyceraldehyde triphosphate. These pathways preferentially provide substrates for different classes of terpenoids [8]. Typically, the MVA pathway is responsible for the synthesis of sesquiterpenes, while monoterpenes are synthesized via MEP-derived precursors in plastids. However, recent studies suggest that these pathways are interconnected through species- and tissue-specific metabolic cross-talk [9], providing additional possibilities for terpenoid biosynthesis.

The monoterpene geraniol, known for its sweet, rose-like aroma, is prevalent in floral scents and has been extensively applied in the cosmetic and fragrance industries. Geraniol is synthesized from geranyl diphosphate (GPP), the direct product of IPP and DMAPP condensation. Two primary pathways have been proposed for geraniol formation. Typically, geraniol is synthesized within plastids by geraniol synthase (GES), which belongs to the terpene synthase (TPS) family [10]. This enzyme utilizes GPP sourced from the MEP pathway in plastids and operates through a carbocation mechanism [8, 11] (hereafter referred to as the MEP-TPS case). This process was first identified in snapdragon flowers [8] and was later confirmed in various plant species [12]. Interestingly, a recent study in several Rosaceae species demonstrated that geraniol can also be produced from GPP derived from the cytosolic MVA pathway [13]. In this case, a cytosolic nudix hydrolase (NUDX) directly hydrolyses GPP to geraniol phosphate, which in turn is dephosphorylated by phosphatase to geraniol [14] (hereafter referred to as the MVA-NUDX case). A transgenic study in *Nicotiana benthamiana* indicated that the cytosolic potential for geraniol biosynthesis mainly relies on the local GPP pool and the abundance of GES [15]. These studies collectively suggest a possible alternative cytosolic route for geraniol biosynthesis, utilizing MVA-derived GPP and the TPS GES (hereafter referred to as the MVA-TPS case). However, almost all GES enzymes characterized thus far localize to plastids [12, 16–19], raising questions about the existence of a cytosolic MVA-TPS pathway for geraniol synthesis.

In this study, we used the popular ornamental cactus *E. oxypetalum* as a model system to investigate the emission dynamics and biosynthetic route of its floral scents. We demonstrated the temporal variation in scent during the transient nocturnal flowering period and confirmed that both petals and sepals were the major sources of floral VOCs. By combining transcriptomic and biochemical approaches, we identified and characterized a cytosolic GES *in vitro*. Pathway-specific inhibitor assays revealed that the MVA pathway mainly supplied the substrates for geraniol biosynthesis. In addition, the quantification of metabolites indicated that starch served as the carbon source for the synthesis of terpenoid volatiles. Together, our results confirmed the existence of the MVA-TPS pathway for geraniol biosynthesis in *E. oxypetalum*, broadening our understanding of floral volatiles, especially in the highly specialized cactus family.

## Results

### Geraniol dominates the floral scent of *E. oxypetalum*

**Figure 1:**
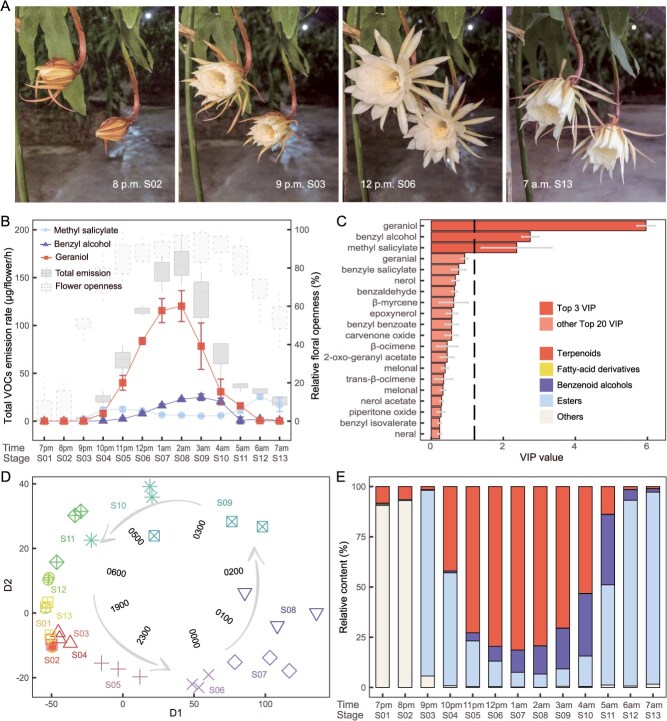
Temporal patterns of flower opening and VOC emission dynamics in *E. oxypetalum.* A: Time-lapse photos showing the rapid blooming of *E. oxypetalum* flowers. B: VOC emission rates of geraniol, benzyl alcohol, methyl salicylate, and total emission across all sampling stages. C: Variable importance in the projection (VIP) values of the top 20 VOCs from OPLS-DA analysis. The three major VOCs with VIP values higher than the threshold are marked. D: Principal components analysis (PCA) clustering of samples according to floral VOC content. The dots are shaped and coloured according to the sampling stage, and the time tags inside the arrow rings correspond to the sampling times of nearby dots. E: Relative concentrations of terpenoids, fatty acid derivatives, benzenoid alcohols, esters, and other VOCs across all the sampled stages. Note that the legend is presented in c.

Unlike many other plant species that bloom gradually over an extended period, *E. oxypetalum* flowers rapidly bloom within a few hours. In our experiments, the flowers typically began opening after 8 p.m. local time and reached full bloom at ~11:00 p.m. (Fig. 1A and B; Movie S1). To investigate the temporal patterns of floral scent emission from *E. oxypetalum*, we collected floral VOCs at 13 different stages using a dynamic headspace collection approach, covering the entire flowering period from 7 p.m. to 7 a.m. the next morning (Fig. 1B and E). In total, we identified 49 VOCs, including 26 terpenoids, 5 fatty acid derivatives, and 18 benzenoids (Table S1). Among them, geraniol, benzyl alcohol, and methyl salicylate were the dominant VOCs, collectively constituting 90.25% of the total emission (165.45 μg/flower/h) at the peak emission stage (2 a.m. local time, Stage 08) (geraniol, 72.54%; benzyl alcohol, 13.72%; methyl salicylate, 4.55%) (Fig. 1B, Table S2). These three VOCs were also key variables in distinguishing between the blooming and nonblooming stages, as revealed by Orthogonal Partial Least Squares Discriminant Analysis (OPLS-DA) (Fig. 1C).

The total floral VOC emissions followed a unimodal pattern, in synchrony with flower openness (Fig. 1B and D). However, different compounds or compound classes exhibited varying temporal patterns. Initially, benzenoid esters dominated, accounting for 92.46% of the total emission shortly after flower opening at 9 p.m. (Stage 03). Terpenoid emission began at ~8 p.m. (Stage 04) and became the dominant VOC class at 1 a.m. (Stage 07), constituting 81.33% of the total emissions. As terpenoid emission increased, benzenoid alcohols gradually surpassed benzenoid esters as the primary benzenoid VOCs, reaching their peak emission at 3 a.m. (Stage 09), slightly after the terpenoid peak. As the flowers subsequently started to wither, the emission of terpenoids and benzenoid alcohols decreased. However, from 5 a.m. (Stage 11), esters constituted the majority of VOCs until the flower fully withered (Fig. 1E). Notably, geraniol and benzyl alcohol displayed a unimodal emission pattern, whereas methyl salicylate exhibited a bimodal pattern (Fig. 1B).

### The floral scent is emitted mainly from the petals and sepals of *E. oxypetalum*

**Figure 2:**
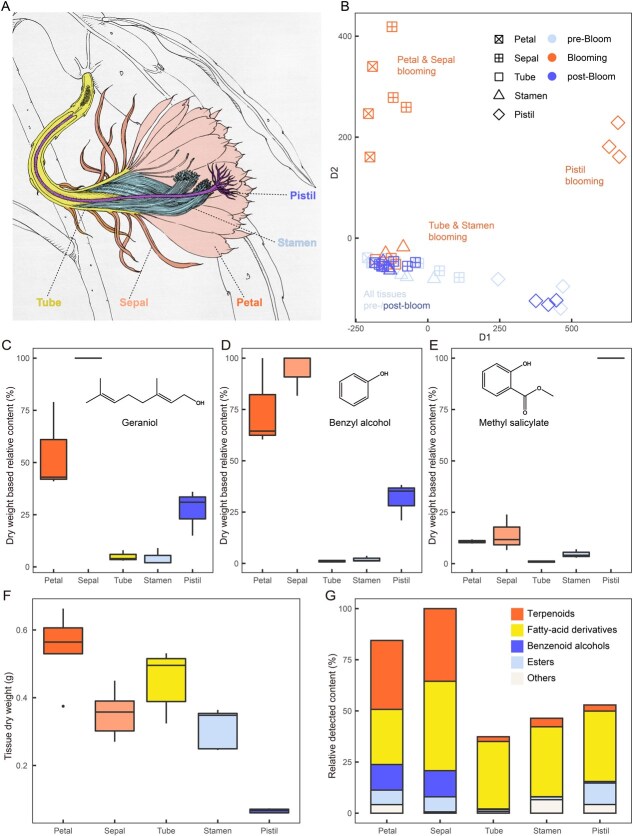
Emission patterns of five floral tissues during the prebloom, blooming, and postbloom stages. A: Schematic diagram of the longitudinal section of *E. oxypetalum* floral tissues, with the five types of tissues coloured differently. B: PCA of the VOC blends emitted from peals, sepals, floral tubes, stamens, and pistils at the prebloom, blooming, and postbloom stages. C–E: Dry weight-based contents of geraniol, benzyl alcohol, and methyl salicylate in five floral tissues at the blooming stage. F: Dry weight of five tissues sampled at the blooming stage, showing pistil contributes little to overall VOCs emission. G: Relative content of five major classes of VOCs at the blooming stage, not adjusted by dry mass, showing petal and sepal contribute largely to overall emission.

To determine the primary source for floral VOC biosynthesis and emission, we analysed the VOC profiles of five organs, namely, petals, sepals, floral tubes, stamens, and pistils (Fig. 2A), at three flower developmental stages, prebloom (S1), blooming (S8), and postbloom (S13), via solid-phase microextraction. The VOC blends varied substantially depending on the flower developmental stage and the specific flower organ. During the blooming stage, the VOC profiles from petals and sepals were grouped together, whereas pistils formed a distinct cluster. Additionally, stamens and tubes constituted another cluster. In contrast, before and after the blooming period, these distinctions were less pronounced, with pistils being the only group that remained separated from the others (Fig. 2B, Table S3). Geraniol and benzyl alcohol were emitted primarily from petals and sepals, followed by pistils, floral tubes and stamens, whereas methyl salicylate was emitted mainly from pistils, followed by sepals and petals (Fig. 2C–E). Considering that petals and sepals accounted for the majority of the whole flower biomass, these organs, particularly petals, served as the primary source for geraniol emission (Fig. 2F and G). Therefore, our subsequent analyses focused on geraniol biosynthesis and subcellular compartmentalization in petals.

### Cytosol-localized terpene synthase is responsible for geraniol biosynthesis

A total of 242.13 million reads were generated from nine libraries, resulting in the assembly of 164 996 unigenes and 299 340 transcripts. The average contig N50 was 2339 bp. A total of 64.20% of the transcripts were annotated with the UniProt and nr databases. A total of 45.52% of the transcripts were functionally tagged using the GO database. Trimmed mean of M-values (TMM) were utilized for gene expression quantification.

**Figure 3:**
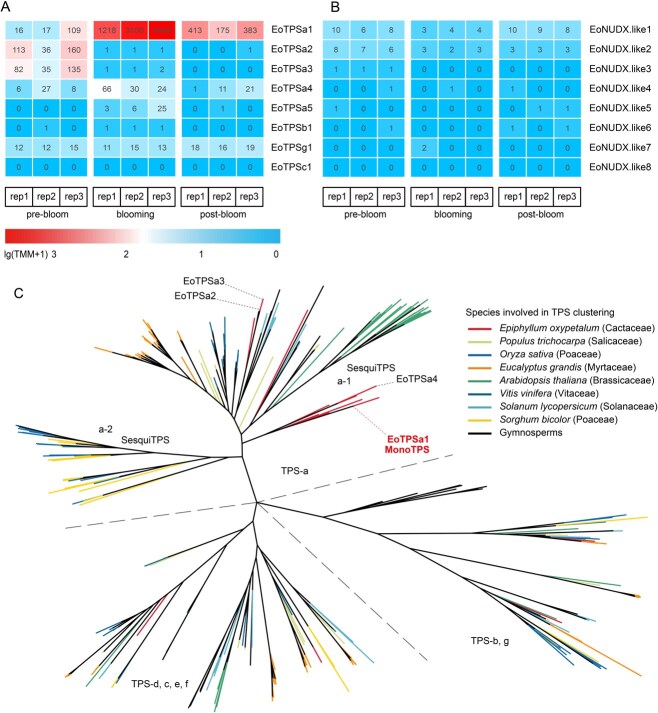
Identification of the TPS for geraniol biosynthesis. A, B: Expression patterns of eight TPSs and eight NUDXs identified with significant expression during the prebloom, blooming, and postbloom stages, with each block coloured according to the TMM value. C: Clustering of identified *E. oxypetalum* TPSs with TPSs from other plant species. The major TPSs in *E. oxypetalum* petals are marked with dashed lines.

To identify the TPSs responsible for geraniol biosynthesis, we focused on transcripts annotated with Pfam TPS models. A total of 84 unique peptides from 23 unigenes were identified as putative TPSs. Sequence clustering analysis of these 23 putative TPSs from *E. oxypetalum* and 342 TPS sequences (Table S5) from nine other species yielded seven subfamilies, with 17 *E. oxypetalum* TPSs belonging to subfamily-a, 1 TPS to subfamily-b, 1 to subfamily-c, 1 to subfamily-e, and 3 to subfamily-g (Fig. 3C, Fig. S7). Among all the putative TPSs identified, the transcript *DN1377.2.1.2* from the subfamily-a exhibited significantly increased expression levels. This particular transcript, designated *EoTPSa1*, was upregulated ~63-fold during the blooming stage, which coincided with the peak emission of geraniol. Apart from this highly expressed transcript, *EoTPSa2, EoTPSa3, EoTPSa4, EoTPSa5*, and *EoTPSg1* also showed some level of expression (Fig. 3A).

The predicted sequence of *EoTPSa1* has an open reading frame (ORF) of 1638 bp, resulting in a protein with 545 amino acids and a molecular weight of 63.29 kDa. The alignment of this protein with other functionally validated GESs revealed the presence of conserved aspartate-rich DDxxD and NSE/DTE motifs, which are important for magnesium-dependent prenyl diphosphate ionization via Class-I mechanisms for TPSs (Figs S2 and S3). Protein structure prediction revealed the characteristic α-β domain structure of monoterpene synthases. Key amino acid residues, such as Asp^293^, Asp^289^, and Glu^443^, were found to be appropriately positioned for the binding of magnesium ions (Fig. S2). The alpha helices in the α domain formed a porous pocket that facilitated substrate binding and enabled water quenching of carbon cation intermediates for geraniol formation. Molecular docking simulations accurately positioned magnesium ions and geraniol diphosphate in the active centre of the enzyme.

**Figure 4:**
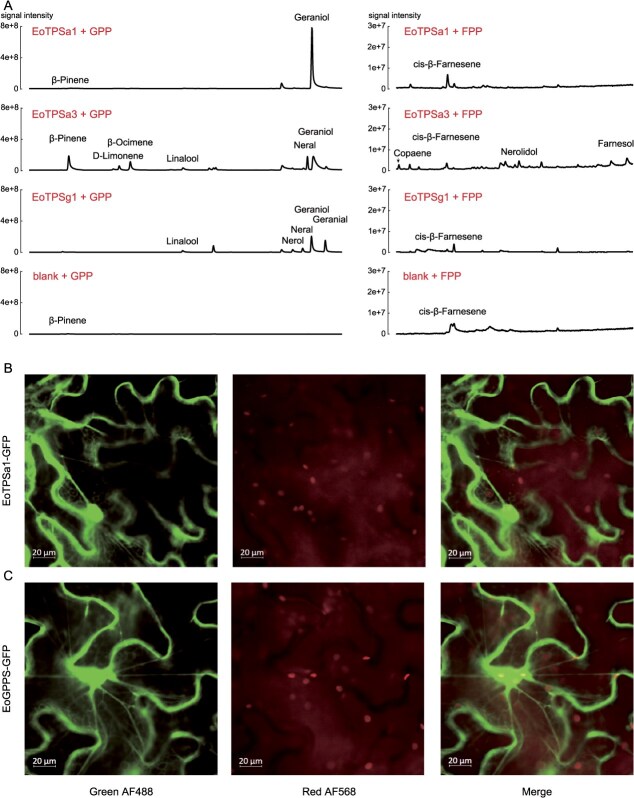
Biochemical characterization and localization of EoTPSa1 as a GES. A: GC–MS chromatograms of the reaction products from *in vitro* incubation of the three GES candidates EoTPSa1, EoTPSa3, and EoTPSg1 with GPP and FPP. Chromatograms in the bottom represent blank controls. Compound names are marked for major peaks. B, C: Subcellular localization of EoTPSa1-GFP and EoGPPS-GFP. Confocal images were taken using the AF488 green band for GFP fluorescence and the AF568 red band for chloroplast autofluorescence.

As demonstrated above, the floral scent of *E. oxypetalum* consists of a variety of terpenes, dominated by geraniol. To characterize the enzymatic origin of these different terpenes, especially geraniol, we attempted to purify these TPSs for *in vitro* enzymatic assays. The full-length sequences for EoTPSa1, EoTPSa3, and EoTPSg1 were amplified, which, along with blank controls, were tested with GPP and farnesyl diphosphate (FPP), precursors for the biosynthesis of all monoterpenes and sesquiterpenes (Fig. 4A). EoTPSa1 showed high specificity and activity for geraniol production, besides a weak farnesene-producing ability. EoTPSa3 produced several monoterpenes such as pinene, ocimene, linalool, and geraniol, as well as several sesquiterpenes such as copaene, farnesene, and farnesol, but all of them were produced in small amounts. EoTPSg3 showed the ability for geraniol, nerol, citral, and neral production, but it could not catalyse sesquiterpene formation at all. Taken together, our *in vitro* enzymatic analyses demonstrate that both EoTPSa1 and EoTPSa3 are putatively bifunctional enzymes, with EoTPSa1 mainly possessing the GPP activity. Although all three enzymes tested shown geraniol-producing potential, eoTPSa1 showed significantly higher expression than others. Thus, we identified EoTPSa1 as the major GES in *E. oxypetalum* during flowering.

Previous works usually identifies enzymes from TPS-b/g subfamilies as GES, which are generally localized in chloroplasts. We therefore predicted cytosolic localization of all TPSs identified with WoLF PSORT and other programmes. Interestingly, EoTPSa1 localizes in nucleus and cytosol, unlike other GESs identified in precious studies (Table S6). We further validated the cytosolic localization of EoTPSa1 through a *Nicotiana* transient expression system (Fig. 4B). We detected strong green fluorescent protein (GFP) signals in the cytosol of the transformed leaf epidermis for the EoTPSa1-GFP and pure GFP constructs. The GFP signal and the red chloroplast fluorescence distribution did not exhibit overlapping patterns, suggesting the absence of protein-sorting activities into chloroplasts. These findings distinguish EoTPSa1 from other characterized GESs, which are typically localized in plastids.

To assess whether other gene families also contribute to geraniol biosynthesis, homologues for the rose geraniol-synthesizing NUDX were identified based on sequence similarity (Fig. S4). Among all the homologues identified, none exhibited significant expression during flowering (Fig. 3B). In addition, most homologues showed a low similarity to rose NUDX. Therefore, we conclude that a cytosol-localized TPS, eoTPSa1, is the major GES in *E. oxypetalum*.

### The MVA pathway provides precursors for geraniol biosynthesis

**Figure 5:**
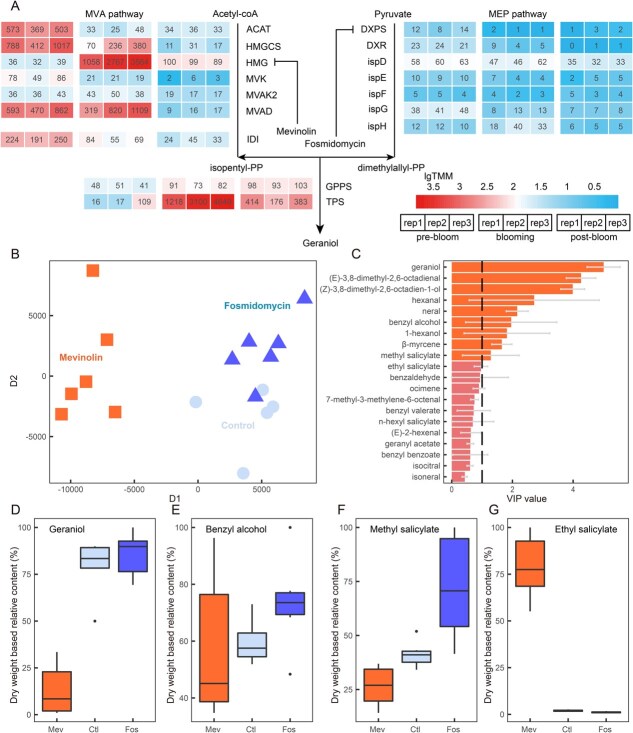
Identification of the major biosynthetic pathway for geraniol biosynthesis in *E. oxypetalum* petals. A: Expression patterns of MVA and MEP pathway genes during the prebloom, blooming, and postbloom stages, with each block coloured according to the TMM value. B: PCA clustering of VOC contents in petals from the mevinolin and fosmidomycin treatment groups and the control group at blooming. Dots were shaped and coloured according to different treatments. C: VIP values of the top 20 VOCs from OPLS-DA analysis. VOCs with VIP values higher than the threshold are marked in different colour. D–G: VOC contents of geraniol, benzyl alcohol, methyl salicylate, and ethyl salicylate in the petals of the mevinolin, fosmidomycin, and control groups.

Next, we questioned whether precursors for geraniol are synthesized in cytosol, instead of having a traditional plastid origin. To investigate the biosynthetic origin of the geraniol precursors IPP and DMAPP, we examined the expression of genes involved in the MVA and MEP pathways. Interestingly, homologous genes from the MVA pathway were upregulated at the blooming stage, in contrast to the uniformly low expression of MEP pathway genes across all three time points (Fig. 5A, Table S8). Specifically, the *HMG* gene, which encodes a putative hydroxymethylglutaryl-CoA reductase and acts as the rate-limiting enzyme of the MVA pathway, was upregulated 69-fold at the blooming stage compared with the prebloom stage. Another gene, *MVAD*, which encodes a putative diphosphate-mevalonate decarboxylase, also exhibited 2-fold upregulation. On average, during the blooming stage, the expression of MVA pathway genes was 33 times greater than that of the MEP pathway genes. To further investigate the predominant role of the MVA pathway in the biosynthesis of geraniol, we employed the pathway-specific inhibitors mevinolin for *HMG* and fosmidomycin for *DXPS* to selectively block the MVA and MEP pathways, respectively. We then collected samples from petals and sepals for VOC characterization. While the VOCs of the fosmidomycin-treated flowers and untreated flowers were similar, there was a noticeable difference in the VOC components of the mevinolin-treated flowers (Fig. 5B and C; Table S4). This distinction was driven mainly by the emission of monoterpenes, particularly geraniol, which showed an 83.4% decrease in mevinolin-treated flowers but a mild increase in fosmidomycin-treated flowers (Fig. 5D). In addition, mevinolin-treated flowers emitted greater amounts of ethyl salicylate but lower amounts of benzyl alcohols and methyl salicylate, whereas fosmidomycin-treated flowers emitted greater amounts of benzyl alcohol and methyl salicylate (Fig. 5E–G). These findings suggest that the MVA pathway is essential for geraniol biosynthesis and that blocking either the MVA or MEP pathway might divert the carbon flow to other biosynthetic pathways, such as the benzenoid biosynthetic pathways.

After IPP and DMAPP are synthesized, they are condensed into IPPs. We further identified IPP synthases in *E. oxypetalum* based on sequence homology. Three transcripts were identified as IPP synthase (Fig. S8), characterized by FARM and SARM catalysing motifs, one of which showed the high similarity to a known GPP synthase (Fig. S5), with the DDVLD motif at both FARM and SARM (thereafter coined as EoGPPS). The other two synthases featured DDIMD and DDYLD at the FARM and SARM motifs, respectively, a signature for FPP synthases [20] (thereafter coined as EoFPPS1 and EoFPPS2, respectively) (Fig. S6). All these three enzymes were predicted to have cytosolic localization (Table S6), but only EoGPPS showed the significant expression (Fig. 5A). We also validated the cytosolic localization of EoGPPS with *Nicotiana* transient expression system (Fig. 4C). Collectively, our study points out that all steps of geraniol biosynthesis in *E. oxypetalum* are localized in cytosol.

### Amyloplast degradation coincides with volatile emission during flowering

**Figure 6:**
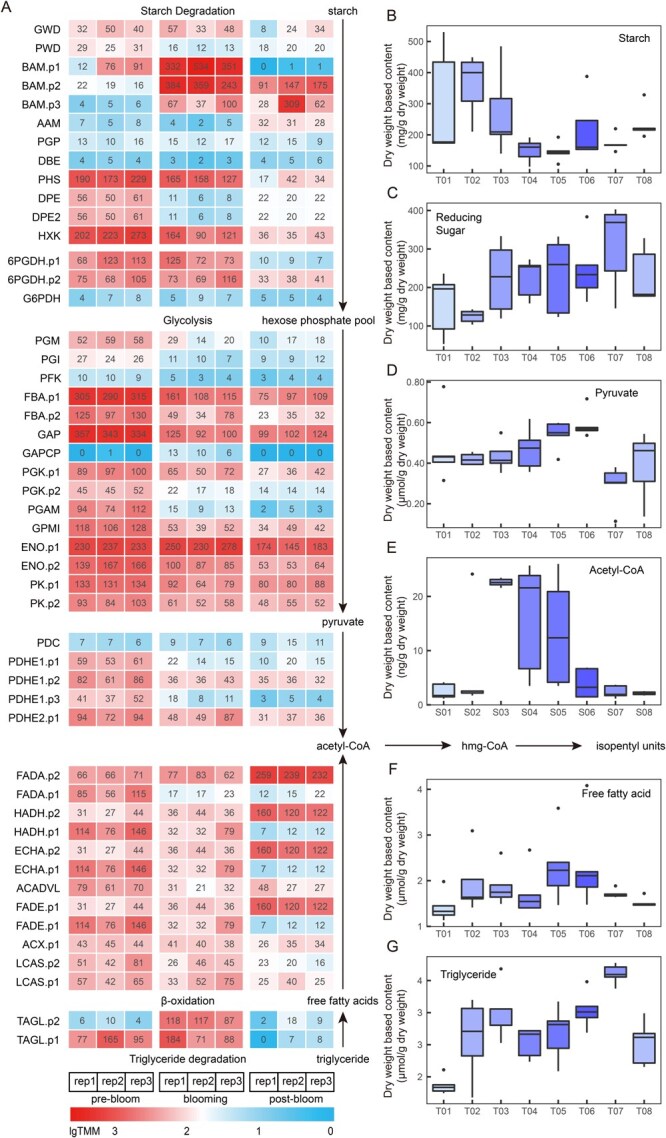
Correlation of geraniol biosynthetic pathway and carbon source in *E. oxypetalum* petals. A: Expression patterns of starch degradation, glycolysis, and β-oxidation pathway genes at prebloom (T03), blooming (T06), and postbloom (T07), with each block coloured according to the TMM value. B–G: Concentrations of starch, reducing sugars, pyruvate, acetyl-CoA, free fatty acids, and triglycerides in petals from sampling stage T01 to T08.

To further investigate floral VOC metabolism in *E. oxypetalum*, we investigated the expression patterns of homologous genes involved in starch degradation, glycolysis, triglyceride degradation, and fatty acid β-oxidation. These genes presented inconsistent expression patterns during the prebloom stage compared with the blooming and postbloom stages. Notably, the beta amylase *BAM*, which catalyses the initial step of starch degradation, was upregulated 6.8-fold at the full-blooming stage, whereas the genes involved in triglyceride degradation and fatty acid β-oxidation did not exhibit a similar trend (Fig. 6A, Table S8).

Biochemical quantification revealed that starch degradation began in the T02–T03 stage, leading to the accumulation of reducing sugars and a sharp increase in acetyl-CoA content, which serves as a direct substrate for the MVA pathway. Over the next half day from T03 to T05, as metabolic activity intensified, starch degradation further increased; the concentration of reducing sugars remained consistently high, while the acetyl-CoA content progressively declined. Although the total amount of pyruvate was relatively low, it exhibited a significant upwards trend, indicating increased metabolism. By the T05–T06 stage, the starch concentration reached its lowest level, the content of pyruvate reached its peak, and acetyl-CoA was depleted to its initial level (Fig. 6B–E). However, not only were triglycerides and free fatty acids detected slightly above the detection threshold from T01 to T07, but the trend of their changes was also independent of acetyl-CoA (Fig. 6F and G). Together, these findings suggest that amyloplast degradation coincides with rapid volatile biosynthesis and emission. However, the exact carbon source of volatile biosynthesis requires further validation.

## Discussion

The biosynthesis and emission patterns of floral volatiles are highly diverse among different taxa. The Cactaceae family contains several well-known ornamental species because of their unique morphological characteristics and attractive flowers [21]. Extensive work has focused on their pollination mode and ecological relevance, but the biosynthesis of their floral scents remains mostly unexplored. Here, using the famous *E. oxypetalum* as a model system, we investigated the composition and biosynthesis of its floral scents in great detail. We first characterized its highly dynamic scent composition and then identified petals and sepals as the major sources of floral scent. By exploring the biosynthesis of its major component geraniol, we identified a novel MVA-TPS for geraniol biosynthesis in *E. oxypetalum*.

### Highly dynamic composition of *E. oxypetalum* floral scents during transient blooming

*Epiphyllum oxypetalum* is renowned for its impressively large flowers and strong scents, traits that are emblematic of the Cactaceae family. Previous investigations categorized cactus flowers into different classes according to their pollination mode, each with distinct floral VOC compositions and emission dynamics [4]. Like nocturnal blooming cacti, *E. oxypetalum* emits excessively high contents of terpenoid and benzenoid volatiles during transient blooming, which lasts for only one night. The nocturnal flowering and strong scent emission may both be due to its coevolution with insects [22] and a legacy of drought adaptation when ancient cactus first thrived in the central Andeans [23]. Interestingly, in our study, the composition of the *E. oxypetalum* scent showed significant hour-to-hour differences. Geraniol was the primary compound responsible for its floral scents and exhibited a unimodal emission pattern peaking at full bloom. The two top benzenoids in *E. oxypetalum*, benzyl alcohol and methyl salicylate, exhibited possible substrate competition [24], as benzyl alcohol peaked around full bloom, whereas methyl salicylate peaked before and after full bloom. The biosynthesis of these VOCs primarily occurred in petals and sepals, both of which have high surface-to-volume ratios, to promote overall scent emission. Additionally, geraniol, benzyl alcohol, and methyl salicylate are known to convey chemical signals between plants and insects, especially geraniol for hawk-moth [25]; thus, they may play crucial roles in the interaction between *E. oxypetalum* and pollinators. Whether these compounds, either individually or in specific ratios, convey specific chemical messages to pollinators during flowering remains to be investigated [26].

### Geraniol biosynthesis via the MVA-TPS pathway

Generally, monoterpenes are synthesized with plastid-localized monoterpene synthase via MEP-derived GPP (MEP-TPS case) [8]. Interestingly, recent work in rose has revealed a special NUDX that catalyses the hydrolysis of GPP into geraniol (MVA-NUDX case) [13, 14]. To date, only a few studies have reported that NUDX is capable of hydrolysing GPP into geraniol in rose hybrids and *Pelargonium graveolens* [27], whereas both the TPS and MVA pathways are widely distributed in higher plants. However, in addition to these two canonical cases, several recent reports have provided several exceptions. For instance, several cytosolic-localized GESs have been characterized in *Fragaria ananassa* [28] and *Lippia dulcis* [29], and a possible GPP pool in the cytosol has been suggested in *N. benthamiana* [15, 30] and *Solanum lycopersicum* [31, 32]. Moreover, isotope labelling experiments have shown MVA-dependent linalool formation in raspberry, along with MEP-dependent sesquiterpene formation in snapdragon [33]. These results suggest a possible new route for monoterpene biosynthesis, in which a cytosolic TPS might utilize MVA-derived GPP for monoterpene production (MVA-TPS case).

In the present study, we provided both biochemical and transcriptomic evidence that cytosolic-localized TPS utilized MVA-derived GPP to synthesize geraniol in *E. oxypetalum* flowers. The cytosolic localization of both GPP synthase and GES, as well as the significant reduction of terpenoid content during the mevinolin treatment, provided compelling evidence supporting the MVA-TPS pathway for geraniol production. As previously summarized [10], subfamily-a TPSs catalyse sesquiterpene biosynthesis in cytosol, while subfamily-b/g TPSs prefer monoterpene substrate in chloroplasts. Yet our study has proved that EoTPSa1 and EoTPSa3, both belonging to the subfamily-a, are bifunctional TPSs capable of using both GPP and FPP as substrate, while EoTPSg1, a subfamily-g TPS, can only catalyse the formation of monoterpenes. Specifically, we found that EoTPSa1 efficiently synthesized geraniol as the dominant monoterpene, with small amounts of farnesene slightly above the detection limit. EoTPSa3 preferentially catalysed the formation of several monoterpenes (e.g. geraniol, pinene, ocimene, and linalool) and sesquiterpenes (e.g. farnesene and farnesol), while EoTPSg1 synthesized solely several monoterpenes, including geraniol, nerol, citral, and neral. The diversity of compounds synthesized by these three TPSs could partially explain the complex temporal evolution of the terpenoid composition of *E. oxypetalum* scents during the short blooming period. Although EoTPSa1 is a bifuncitonal enzyme, it shows a much stronger catalytic activity towards GPP compared to FPP, since monoterpens, especially geraniol, are the predominant reaction products from *in vitro* incubation of EoTPSa1 with GPP and FPP. Moreover, although EoTPSa1, EoTPSa3, and EoTPSg1 are all able to catalyse the formation of geraniol, the geraniol-synthesizing potential of EoTPSa1 is ~100-fold stronger than that of the other two. Collectively, these findings suggest that EoTPSa1 is the main GES responsible for geraniol biosynthesis in *E. oxypetalum* during flowering.

The observation of the monoterpene synthesis activity of the subfamily-a TPSs is consistent with previous findings concerning active gene neofunctionalization during terpenoid evolution and the preference for utilizing existing enzymes for specialized metabolism with catalytic promiscuity [34]. The incorporation of cytosolic MVA pathways rather than plastid-derived substrates coupled with downstream transportation can significantly improve catalytic efficiency if downstream enzymes are localized in the cytosol [15].

### Starch degradation coincides with the synthesis of floral VOCs

The MVA pathway uses acetyl-CoA as a substrate, which can be derived from both starch and lipid degradation. Transcriptome assembly and quantification revealed active expression at the blooming stage for both β-amylase (BAM) and TAGL, the initial steps of these two degradation pathways. Biochemical quantification revealed excessively high contents of starch (up to 30% dry weight) and reducing sugars in petals but an almost complete lack of triglycerides. A significant decrease in starch content coincides with flower blooming. The decrease in starch content suggests that starch degradation begins in the morning on the day of blooming, creating a reservoir of substrates (free sugar, acetyl-CoA) before flowering, which in turn can be used by the active metabolism during flower opening and volatile biosynthesis. However, our results do not provide unambiguous evidence for the carbon flow during flower opening. To make a solid conclusion, future studies should track the carbon flow during flowering, particularly those directed to floral VOC biosynthesis.

Taken together, our findings suggest that an adequate supply of substrates, the strict regulation of enzymes, and the selection of the cytoplasm as the site for geraniol synthesis are the key factors that enable *E. oxypetalum* to release large quantities of volatile compounds, especially geraniol, within its brief flowering period. These features distinguish *E. oxypetalum* apart from other cacti, which typically have longer flowering durations and emit few fragrances. The unique explosive geraniol synthesis strategy of *E. oxypetalum* not only advances our fundamental understanding of volatile biosynthesis mechanisms and desert environment adaptation strategies for plants in the cactus family, but also offers potential applications in the horticultural and perfume industries.

## Materials and methods

### Plant materials and chemicals

Plant materials of *E. oxypetalum* were collected from 5-year-old cottage seedlings at the *E. oxypetalum* planting demonstration base of Sichuan Yuanlan Agricultural Development Co., Ltd. in Zizhong, Sichuan, China (29°40′N, 104°45′E). The seedlings were grown under seminatural conditions with pine needle mulch soil. To aid in the development of healthy flowers, a specialized phosphate–potash–amino acid mixture was applied as a foliar fertilizer. Shaded roofs were used during summer to protect plants from excessive sunlight. For RNA-seq, quantitative real-time polymerase chain reaction (qRT-PCR), molecular cloning, and biochemical experiments, samples from various parts of the flowers were collected in September, immediately frozen in liquid nitrogen and stored at −80°C. The materials for floral organ VOC analysis using solid-phase micro extraction (SPME) were stored in glass bottles, immediately frozen, and stored on dry ice. Floral scent collections from the whole flower were conducted *in situ* in October using a dynamic headspace sampling approach. All chemical reagents used were purchased from Shanghai Aladdin Biochemical Technology Co. Ltd. (www.aladdin-e.com).

### Floral VOC collection and GC–MS analysis

For *in situ* VOC collection, floral scents were sampled hourly from 7 p.m. to 7 a.m. (Fig. S1) the following day via a push–pull headspace sampling system. Briefly, precleaned (120°C for 1 h) polyethylene terephthalate (PET) bags (35 × 43 cm) were securely attached to the pedicel to enclose the flower. Filtered air was circulated through the bag at a flow rate of 1000 ml min^−1^ via a battery-operated pump connected to Teflon tubing. The incoming air was purified by an activated charcoal filter to remove particles and VOCs, and a copper tube was coated with potassium iodide to prevent ozone. Air was removed from the bag at a flow rate of 200 ml min^−1^ through a stainless steel adsorbent cartridge packed with 155 mg of Tenax TA, 66 mg of Carbopack B, and 75 mg of Carbopack X (mesh 60/80; Labinno Application Technology Co., Ltd, Guangzhou, China). VOCs were collected from three flowers simultaneously, each from a different plant. After sampling, the cartridges were sealed with Teflon-coated brass caps and stored at 4°C until analysis. Blank measurements from empty PET bags were performed to characterize impurities originating from the sampling or analysis system.

VOCs trapped in adsorbent cartridges were thermally desorbed (TD100-xr, Markes International Ltd., Llantrisant, UK) at 250°C for 10 min and analysed via gas chromatography–mass spectrometry (GC–MS; 8890 GC, 5977B MSD, Agilent Technologies, Santa Clara, CA, USA). The VOCs were first cryofocused at −10°C and then injected into an HP-5MS capillary column (30 m × 0.25 mm, film thickness 0.25 μm) using helium as the carrier gas at a flow rate of 1.0 ml min^−1^ using the following temperature programme: 40°C, held for 1 min; raised at 5°C min^−1^ to 210°C, raised at 20°C min^−1^ to 250°C, held for 8 min.

For flower organ VOC extraction, VOCs were sampled via SPME fibres (SUPELCO 57328-U, PDMS) for 40 min at 50°C. The VOCs adsorbed by the SPME fibres were thermally desorbed at 230°C before analysis via GC–MS (SHIMADZU GCMS-QP2010) with a Rtx-5 column. Helium was used as the carrier gas at a flow rate of 1.0 ml min^−1^ using the following temperature programme: 50°C, held for 3 min; raised at 6°C min^−1^ to 200°C, raised at 10°C min^−1^ to 240°C, and held for 6 min. Chromatograms were analysed via PARADISe v.6.0.1 [35]. The compounds were identified using pure standards when available or were tentatively identified via the NIST20 library. The VOC concentrations in the blanks were subtracted from those in the samples.

### Inhibition assay for the MVA and MEP pathways

In the inhibition experiment, we administered a 100 μM solution of the MEP-specific inhibitor fosmidomycin or the MVA-specific inhibitor mevinolin [8] by uniformly injecting 1 ml of the solution into the junction of petals and perianth tubes at three specific time points: 9 p.m. the day before bloom, 9 a.m., and 7 p.m. The control group was injected with ddH_2_O. Floral scent samples were collected at 12 a.m. the next day (Fig. S1). Throughout the experiment, the chosen inhibitors did not cause any noticeable effects on the appearance of the flowers. For the MVA inhibitor assay, lactone hydrolysis of mevinolin was performed following a previous report [36].

### RNA-seq and analysis

Total RNA was extracted from *E. oxypetalum* petals using an improved hexadecyl trimethyl ammonium bromide (CTAB) method. At each sampling stage, i.e. prebloom (9 a.m.), blooming (S06), and postbloom (S13) (Fig. S1), three replicates of DNBSEQ RNA-seq libraries were prepared and quality checked. The libraries were sequenced via the MISEQ-2000 platform by BGI, resulting in 150-bp paired-end reads. The raw sequencing reads subsequently underwent a quality control and cleaning process [37] involving the removal of adaptors, ambiguous N-containing reads, and low-quality reads. The resulting clean data were then *de novo* assembled into transcripts using Trinity [38, 39].

For sequence annotation, all assembled transcripts were compared to the NCBI Nr, SwissProt, gene ontology (GO), and kyoto encyclopedia of genes and genomes (KEGG) databases with an E-value threshold of 1e^−5^ for BLAST operations [40]. The Pfam protein database was searched via the HMMER programme [41] with an E-value threshold set at 1e^−5^. Annotation integration and functional categorization by GO terms were performed by Trinotate [42]. Later, the abundance estimation and TMM values were calculated using RSEM [43]. Differential expression analysis was conducted using DESeq2 [44], with a false discovery rate (FDR) threshold of <0.01 and an absolute value of the log_2_ ratio ≥ 2 to identify DEGs. GO term enrichment analysis was performed using the GOseq R package [45] on the basis of the Wallenius noncentral hypergeometric distribution. Sequence clustering was carried out using MUSCLE [46], ModelTest-NG [47], and RAxML-NG [48].

For Sequence identification of gene families, Hidden Markov Model (HMM) of these gene families from interproscan (PF01397.2, PF03936.19, PF19083.3 for TPS; PF00293.33 for NUDX) were used as queries to search against *E. oxypetalum* transcriptome assembly using HMMER 3.3. After filtering round1 search results with e-value threshold at 1e–10, the species-specific HMM models for respective gene families were built and then used as queries to search against the transcriptome assemblies. These round2 search results were then filtered with 1e–10 threshold for respective gene family members. For pathway identification, CDS sequences of respective genes were downloaded from KEGG, and searched against the transcriptome assemblies using BLAST. Sequences with high similarity and expression were identified as respective genes.

### qRT–PCR of genes responsible for geraniol synthesis

Specific primers for qRT–PCR were designed with NCBI. Total RNA was extracted from *E. oxypetalum* petals with a Sangon Biotech Plant Total RNA Isolation Kit (Sangon Company, https://www.sangon.com). The full-length cDNA was then reverse transcribed using the Takara Prime Script™ RT reagent Kit (Perfect Real Time) (https://www.Takarabiomed.com.cn). qRT–PCR was performed with a Takara RR430B TB Green Fast qPCR system, with independent RNA preparations used as biological replicates. *Ubqln* gene transcripts were used as controls. The relative expression levels of the target genes were calculated using the 2^-ΔΔCt^ method. The specificity of each primer pair was verified via agarose gel electrophoresis, melting curve analysis, and sequence homology with a local script. All relevant primer sequences are listed in the supplementary materials (Table S7).

### Subcellular localization of candidate genes

Primers for *EoTPSa1* were designed to amplify specific cDNA fragments via the Takara PrimeSTAR® Max PCR system. We utilized SignalP 6.0 for subcellular localization prediction. The transient gene expression in *N. benthamiana* leaves was subsequently performed following established procedures with pSuper1300 and *Agrobacterium tumefaciens* strain GV3101. After infiltration, the plants were kept in the dark for 24 h. Two to five days after infiltration, confocal images of *N. benthamiana* leaves were acquired using Zeiss CELL Observer SD.

### Enzymatic activity validation of EoTPSa1

Structure prediction and molecular docking of EoTPSa1 were performed with Alphafold2 [49] and AutoDock [50], respectively. For *in vitro* enzymatic analysis, the cDNA of *EoTPSa1, EoTPSa3,* and *EoTPSg1* was successfully amplified and ligated into multiple cloning sites of the pET-28a expression vector utilizing Takara In-Fusion Snap Assembly Master Mix. The constructed vector was then transformed into *Escherichia coli Rosetta-gami 2 (DE3)* cells. Positive clone incubation, protein induction, extraction, and purification were conducted using a Bacterial Protein Extraction Kit (Sangon) and Ni-NTA spin columns (Sangon) according to the manufacturer’s instructions. The purified protein was examined by 12.5% (w/v) SDS–PAGE.

The enzymatic assay was conducted in glass inserts sealed inside HPLC vials, preventing product evaporation and plastic contamination. Hexane was added between inserts and HPLC vials for rapid heat transfer. A total of 200 μl of reaction buffer (10 mM Tris–HCl, 10 mM MgCl_2_) were supplemented with 0.4 μM GPP or 0.1 μM FPP, and overlaid with 50 μl hexane for product extraction. A total of 0.5 μg of purified protein was added. After the reaction system was sealed, the mixture was incubated at 25°C for 30 min. The reaction products were then extracted by vigorously shaking for 10 s. Then the reaction is stopped by freezing the mixture on ice. A total of 1 μl of the dehydrated hexane supernatant was injected for GC–MS analysis. Verification and quantification of the reaction products were achieved by comparison with the NIST17 library and authentic standards.

### Biochemical characterization of the acetyl-CoA-generating metabolic pathway

The samples were collected from petals at different time points on T01–T08 (Fig. S1). The following reagents were purchased from Sangon Biotech Company for the biochemical experiments: reducing sugars (D799393), triglycerides (D799795), free fatty acids (D799793), and acetyl-CoA (D751001). Amyl and soluble polysaccharides were separated via centrifugation at 10 000 rpm for 10 min after homogenization. The amylum fraction was washed with ddH_2_O, while soluble polysaccharides were precipitated with 80% ethanol. Both components were then tested with the Amylum Content Assay Kit (D799325). The absorbance of each well was measured at the required wavelength using an Agilent BioTek Synergy H1 Multimode Reader.

## Supplementary Material

Web_Material_uhaf039

## Data Availability

All the data needed to evaluate the conclusions in the paper are presented in the paper and/or the Supplementary Materials. The raw transcriptome reads for the nine individuals in this study have been deposited in the National Genomics Data Center (https://ngdc.cncb.ac.cn) under accession number PRJCA024038.

## References

[1] Dudareva N, Klempien A, Muhlemann JK. et al. Biosynthesis, function and metabolic engineering of plant volatile organic compounds. New Phytol. 2013;198:16–32 23383981 10.1111/nph.12145

[2] Dötterl S, Gershenzon J. Chemistry, biosynthesis and biology of floral volatiles: roles in pollination and other functions. Nat Prod Rep. 2023;40:1901–37 37661854 10.1039/d3np00024a

[3] Zhang W, Jiang Y, Chen F. et al. Dynamic regulation of volatile terpenoid production and emission from Chrysanthemum morifolium capitula. Plant Physiol Biochem. 2022;182:11–21 35453029 10.1016/j.plaphy.2022.03.039

[4] Kaiser R, Tollsten L. An introduction to the scent of cacti. Flavour Fragr J. 1995;10:153–64

[5] Chen W, Viljoen AM. Geraniol — a review of a commercially important fragrance material. South Afr J Bot. 2010;76:643–51

[6] Abbas F, Ke Y, Yu R. et al. Volatile terpenoids: multiple functions, biosynthesis, modulation and manipulation by genetic engineering. Planta. 2017;246:803–16 28803364 10.1007/s00425-017-2749-x

[7] Muhlemann JK, Klempien A, Dudareva N. Floral volatiles: from biosynthesis to function. Plant Cell Environ. 2014;37:1936–49 24588567 10.1111/pce.12314

[8] Dudareva N, Andersson S, Orlova I. et al. The nonmevalonate pathway supports both monoterpene and sesquiterpene formation in snapdragon flowers. Proc Natl Acad Sci. 2005;102:933–8 15630092 10.1073/pnas.0407360102 PMC545543

[9] Vranová E, Coman D, Gruissem W. Structure and dynamics of the isoprenoid pathway network. Mol Plant. 2012;5:318–33 22442388 10.1093/mp/sss015

[10] Chen F, Tholl D, Bohlmann J. et al. The family of terpene synthases in plants: a mid-size family of genes for specialized metabolism that is highly diversified throughout the kingdom: terpene synthase family. Plant J. 2011;66:212–29 21443633 10.1111/j.1365-313X.2011.04520.x

[11] Christianson DW . Structural and chemical biology of terpenoid cyclases. Chem Rev. 2017;117:11570–648 28841019 10.1021/acs.chemrev.7b00287 PMC5599884

[12] Abbas F, Ke Y, Yu R. et al. Functional characterization and expression analysis of two terpene synthases involved in floral scent formation in Lilium ‘Siberia’. Planta. 2019;249:71–93 30218384 10.1007/s00425-018-3006-7

[13] Conart C, Bomzan DP, Huang XQ. et al. A cytosolic bifunctional geranyl/farnesyl diphosphate synthase provides MVA-derived GPP for geraniol biosynthesis in rose flowers. Proc Natl Acad Sci USA. 2023;120:e2221440120 37126706 10.1073/pnas.2221440120 PMC10175749

[14] Magnard J-L, Roccia A, Caissard JC. et al. Biosynthesis of monoterpene scent compounds in roses. Science. 2015;349:81–3 26138978 10.1126/science.aab0696

[15] Dong L, Jongedijk E, Bouwmeester H. et al. Monoterpene biosynthesis potential of plant subcellular compartments. New Phytol. 2016;209:679–90 26356766 10.1111/nph.13629

[16] Bartram S, Jux A, Gleixner G. et al. Dynamic pathway allocation in early terpenoid biosynthesis of stress-induced lima bean leaves. Phytochemistry. 2006;67:1661–72 16580034 10.1016/j.phytochem.2006.02.004

[17] Gutensohn M, Orlova I, Nguyen TTH. et al. Cytosolic monoterpene biosynthesis is supported by plastid-generated geranyl diphosphate substrate in transgenic tomato fruits. Plant J. 2013;75:351–63 23607888 10.1111/tpj.12212

[18] Opitz S, Nes WD, Gershenzon J. Both methylerythritol phosphate and mevalonate pathways contribute to biosynthesis of each of the major isoprenoid classes in young cotton seedlings. Phytochemistry. 2014;98:110–9 24359633 10.1016/j.phytochem.2013.11.010

[19] Mendoza-Poudereux I, Kutzner E, Huber C. et al. Metabolic cross-talk between pathways of terpenoid backbone biosynthesis in spike lavender. Plant Physiol Biochem. 2015;95:113–20 26254184 10.1016/j.plaphy.2015.07.029

[20] Kumar A, Patekar S, Mohapatra S. et al. Isoprenyl diphosphate synthases of terpenoid biosynthesis in rose-scented geranium (*Pelargonium graveolens*). Plant Physiol Biochem. 2024;210:108590 38574692 10.1016/j.plaphy.2024.108590

[21] Mauseth JD . Structure-function relationships in highly modified shoots of Cactaceae. Ann Bot. 2006;98:901–26 16820405 10.1093/aob/mcl133 PMC2803597

[22] Guerrero PC, Majure LC, Cornejo-Romero A. et al. Phylogenetic relationships and evolutionary trends in the cactus family. J Hered. 2019;110:4–21 30476167 10.1093/jhered/esy064

[23] Hernández-Hernández T, Brown JW, Schlumpberger BO. et al. Beyond aridification: multiple explanations for the elevated diversification of cacti in the New World succulent biome. New Phytol. 2014;202:1382–97 24611540 10.1111/nph.12752

[24] Schuurink RC, Haring MA, Clark DG. Regulation of volatile benzenoid biosynthesis in petunia flowers. Trends Plant Sci. 2006;11:20–5 16226052 10.1016/j.tplants.2005.09.009

[25] Foster And SP, Harris MO. Behavioral manipulation methods for insect pest-management. Annu Rev Entomol. 1997;42:123–46 15012310 10.1146/annurev.ento.42.1.123

[26] Raguso RA . Wake up and smell the roses: the ecology and evolution of floral scent. Annu Rev Ecol Evol Syst. 2008;39:549–69

[27] Bergman ME, Bhardwaj M, Phillips MA. Cytosolic geraniol and citronellol biosynthesis require a Nudix hydrolase in rose-scented geranium (Pelargonium graveolens). Plant J Cell Mol Biol. 2021;107:493–510 10.1111/tpj.15304 33949016

[28] Aharoni A, Giri AP, Verstappen FWA. et al. Gain and loss of fruit flavor compounds produced by wild and cultivated strawberry species. Plant Cell. 2004;16:3110–31 15522848 10.1105/tpc.104.023895 PMC527202

[29] Dong L, Miettinen K, Goedbloed M. et al. Characterization of two geraniol synthases from Valeriana officinalis and Lippia dulcis: similar activity but difference in subcellular localization. Metab Eng. 2013;20:198–211 24060453 10.1016/j.ymben.2013.09.002

[30] Wu S, Schalk M, Clark A. et al. Redirection of cytosolic or plastidic isoprenoid precursors elevates terpene production in plants. Nat Biotechnol. 2006;24:1441–7 17057703 10.1038/nbt1251

[31] Davidovich-Rikanati R, Lewinsohn E, Bar E. et al. Overexpression of the lemon basil alpha-zingiberene synthase gene increases both mono- and sesquiterpene contents in tomato fruit. Plant J Cell Mol Biol. 2008;56:228–38 10.1111/j.1365-313X.2008.03599.x 18643974

[32] Zhou F, Pichersky E. The complete functional characterisation of the terpene synthase family in tomato. New Phytol. 2020;226:1341–60 31943222 10.1111/nph.16431 PMC7422722

[33] Hampel D, Swatski A, Mosandl A. et al. Biosynthesis of monoterpenes and norisoprenoids in raspberry fruits (Rubus idaeus L.): the role of cytosolic mevalonate and plastidial methylerythritol phosphate pathway. J Agric Food Chem. 2007;55:9296–304 17907775 10.1021/jf071311x

[34] Weng J-K . The evolutionary paths towards complexity: a metabolic perspective. New Phytol. 2014;201:1141–9 23889087 10.1111/nph.12416

[35] Johnsen LG, Skou PB, Khakimov B. et al. Gas chromatography – mass spectrometry data processing made easy. J Chromatogr A. 2017;1503:57–64. 28499599 10.1016/j.chroma.2017.04.052

[36] Kita T, Brown MS, Goldstein JL. Feedback regulation of 3-hydroxy-3-methylglutaryl coenzyme A reductase in livers of mice treated with mevinolin, a competitive inhibitor of the reductase. J Clin Invest. 1980;66:1094–100 6903572 10.1172/JCI109938 PMC371547

[37] Bolger AM, Lohse M, Usadel B. Trimmomatic: a flexible trimmer for Illumina sequence data. Bioinformatics. 2014;30:2114–20 24695404 10.1093/bioinformatics/btu170 PMC4103590

[38] Grabherr MG, Haas BJ, Yassour M. et al. Trinity: reconstructing a full-length transcriptome without a genome from RNA-Seq data. Nat Biotechnol. 2011;29:644–52 21572440 10.1038/nbt.1883 PMC3571712

[39] Haas BJ, Papanicolaou A, Yassour M. et al. De novo transcript sequence reconstruction from RNA-Seq: reference generation and analysis with Trinity. Nat Protoc. 2013;8:1494–512 23845962 10.1038/nprot.2013.084 PMC3875132

[40] Altschul SF, Gish W, Miller W. et al. Basic local alignment search tool. J Mol Biol. 1990;215:403–10 2231712 10.1016/S0022-2836(05)80360-2

[41] Eddy SR . Accelerated profile HMM searches. PLoS Comput Biol. 2011;7:e1002195 22039361 10.1371/journal.pcbi.1002195 PMC3197634

[42] Duarte GT, Volkova PY, Geras’kin SA. A pipeline for non-model organisms for de novo transcriptome assembly, annotation, and gene ontology analysis using open tools: case study with Scots pine. Bio Protoc. 2021;11:e3912 10.21769/BioProtoc.3912 PMC7952955 33732799

[43] Li B, Dewey CN. RSEM: accurate transcript quantification from RNA-Seq data with or without a reference genome. BMC Bioinformatics. 2011;12:323 21816040 10.1186/1471-2105-12-323 PMC3163565

[44] Love MI, Huber W, Anders S. Moderated estimation of fold change and dispersion for RNA-seq data with DESeq2. Genome Biol. 2014;15:550 25516281 10.1186/s13059-014-0550-8 PMC4302049

[45] Young MD, Wakefield MJ, Smyth GK. et al. Gene ontology analysis for RNA-seq: accounting for selection bias. Genome Biol. 2010;11:R14 20132535 10.1186/gb-2010-11-2-r14 PMC2872874

[46] Edgar RC . MUSCLE: multiple sequence alignment with high accuracy and high throughput. Nucleic Acids Res. 2004;32:1792–7 15034147 10.1093/nar/gkh340 PMC390337

[47] Darriba D, Posada D, Kozlov AM. et al. ModelTest-NG: a new and scalable tool for the selection of DNA and protein evolutionary models. Mol Biol Evol. 2020;37:291–4 31432070 10.1093/molbev/msz189 PMC6984357

[48] Kozlov AM, Darriba D, Flouri T. et al. RAxML-NG: a fast, scalable and user-friendly tool for maximum likelihood phylogenetic inference. Bioinformatics. 2019;35:4453–5 31070718 10.1093/bioinformatics/btz305 PMC6821337

[49] Jumper J, Evans R, Pritzel A. et al. Highly accurate protein structure prediction with AlphaFold. Nature. 2021;596:583–9 34265844 10.1038/s41586-021-03819-2 PMC8371605

[50] Morris GM, Huey R, Lindstrom W. et al. AutoDock4 and AutoDockTools4: automated docking with selective receptor flexibility. J Comput Chem. 2009;30:2785–91 19399780 10.1002/jcc.21256 PMC2760638

