# Supplementary material for: Floral scent emission of Epiphyllum oxypetalum: discovery of its cytosol-localized geraniol biosynthesis
Source: Hortic Res. 2025 Feb 11;12(5):uhaf039. doi: 10.1093/hr/uhaf039 (PMC11997432; doi:10.1093/hr/uhaf039)
Supplement: Web_Material_uhaf039 [file web_material_uhaf039.zip › Supplementary.figures.docx]

**Supplementary material**

**Floral scent emission of *Epiphyllum oxypetalum*: Discovery of its cytosol-localized geraniol biosynthesis**

Yiyang Zhang ^1, 2*^, Yuhan Zhang ^1, *^, Andong Zhang ^1^, Qiurui Tian ^1^, Bin Yang ^1^, Likun Wei ^1^, Wei Wu ^1^, Ting Zhu ^1^, Zhiwei Zhou ^1^, Jiaqi Wang ^1^, Zhibin Liu ^1^, Wei Tang ^3^, Haijun Xiao ^1^, Mingchun Liu ^1^, Tao Li ^1#^, Qun Sun ^1#^

^1^ Key Laboratory of Bioresources and Eco-environment Ministry of Education, College of Life Sciences, Sichuan University, Chengdu, Sichuan, China

^2^ Peking University-Tsinghua University-National Institute of Biological Sciences Joint Graduate Program, Academy for Advanced Interdisciplinary Studies, Peking University, Beijing, China

^3^ Sichuan Academy of Botanical Engineering, Sichuan Academy of Agricultural Sciences, Zizhong, Sichuan, China

Tao Li, tao.li@scu.edu.cn (co-corresponding author)

Qun Sun, qunsun@scu.edu.cn (co-corresponding author)

**
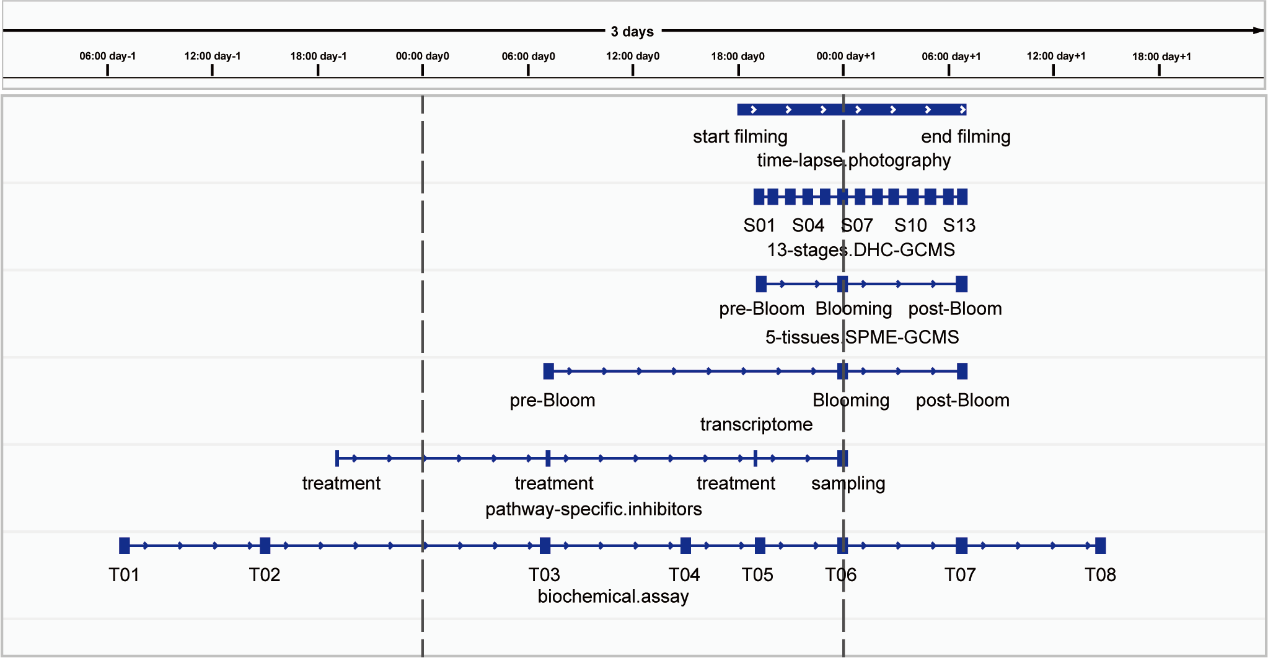
Figure S1. Illustration of sampling time of all experiments in the current study.**

day-1, day0 and day1 indicates the day prior of blooming, the day of blooming and the day after blooming, respectively. Each line indicates a specific experiment. The thicker square indicates time-point for sampling, the narrower square indicates time-point for specific treatment.


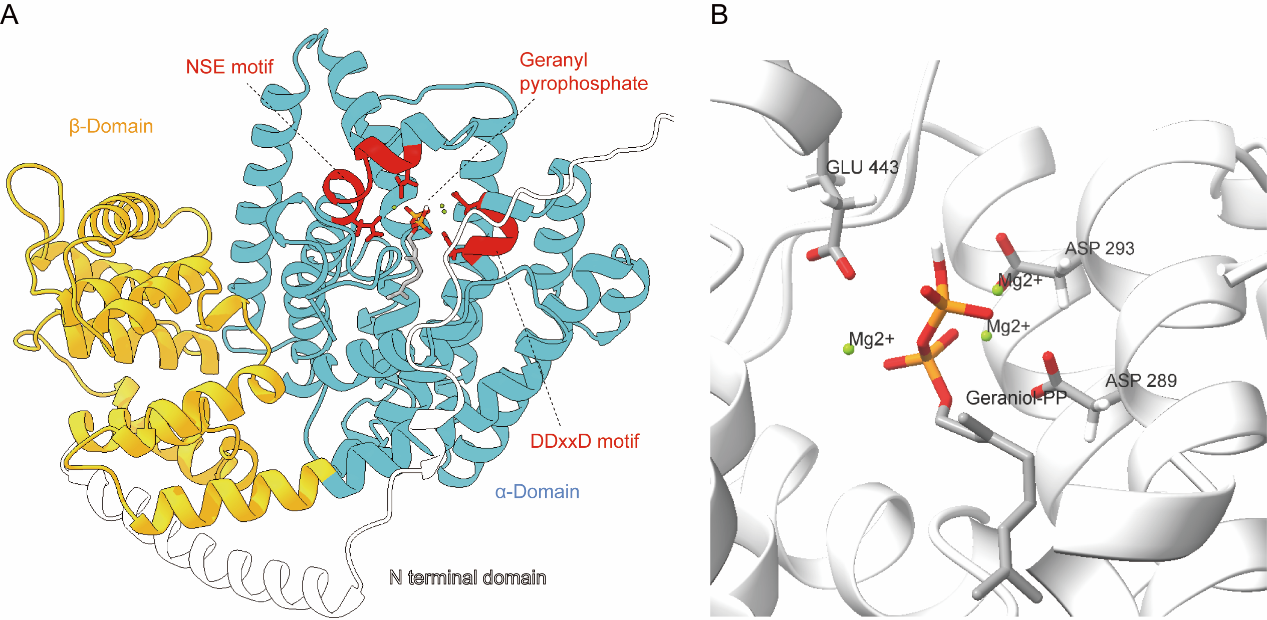


**Figure S2. Protein structure and docking predictions for eoTPSa1**

A: Structure prediction of EoTPSa1 and docking with geraniol diphosphate.

B: detailed illustration of the substrate pocket along with magnesium ions and relevant amino acid residues.

**
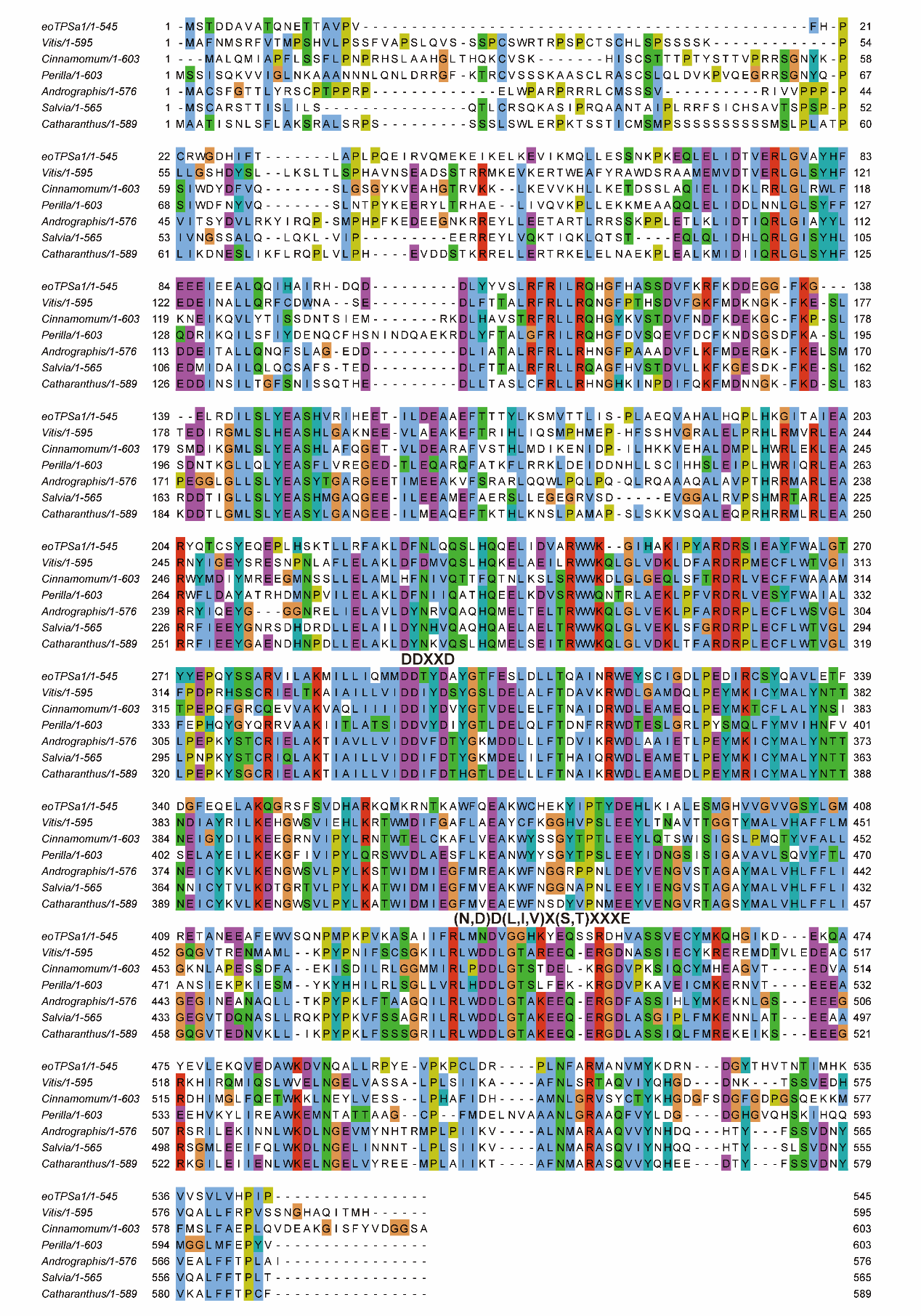
Figure S3. Sequence alignment for geraniol synthases identified**

The sequence of geraniol synthase identified in the current study (EoTPSa1) is aligned to 6 other previously characterized geraniol synthases from *Vitis vinifera* (NP_001267920), *Cinnamomum tenuipile* (CAD29734), *Perilla frutescens* (ABB30218), *Andrographis paniculata* (XP_051134212), *Salvia splendens* (XP_042068265) and *Catharanthus roseus* (AHA82032). Conserved catalytic DDXXD and (N,D)D(L,I,V)X(S,T)XXXE motifs are marked.

**
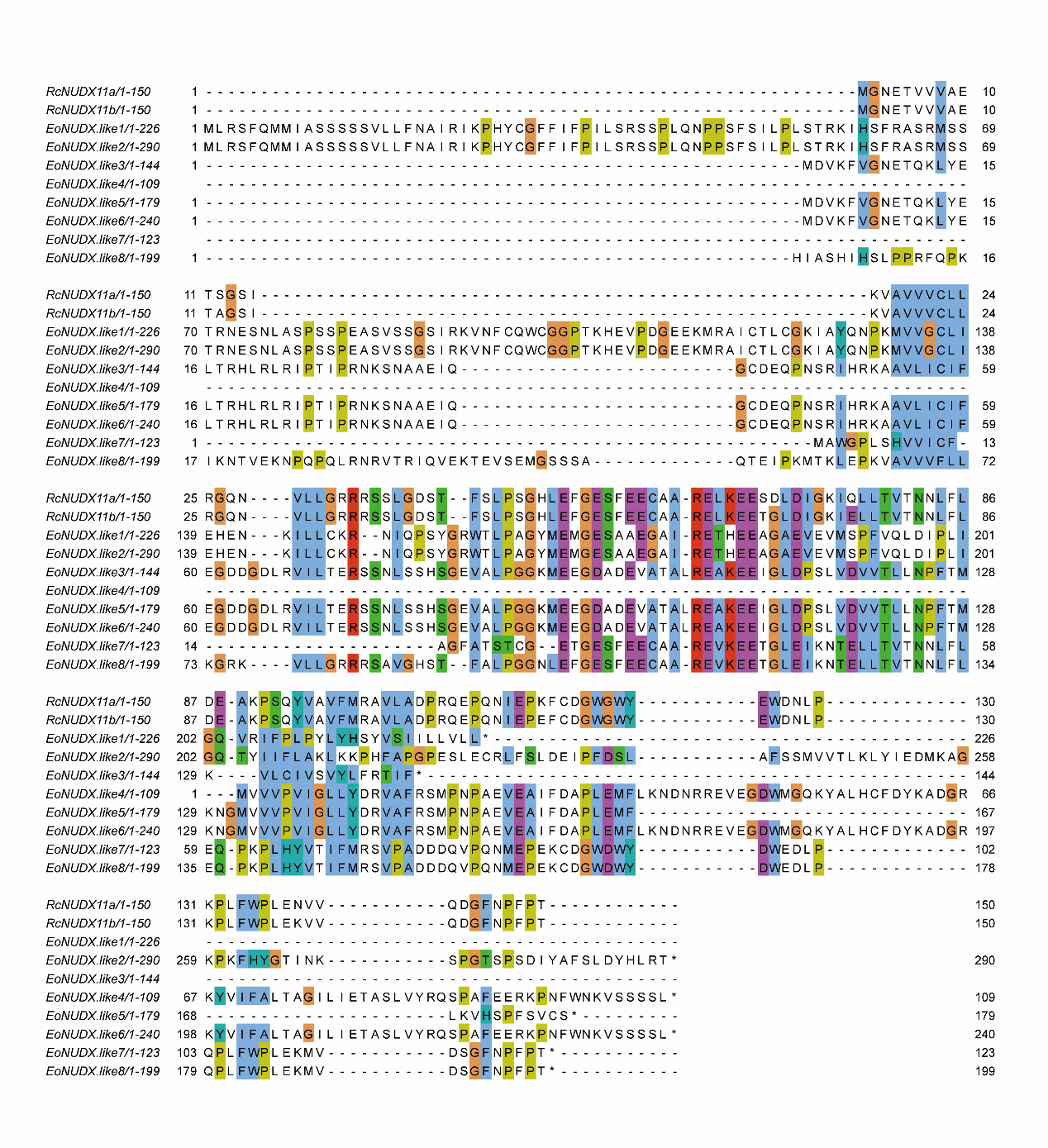
Figure S4. Sequence alignment for nudix hydrolases identified**

The nudix hydrolase-like sequences identified in the current study by sequence homology is aligned to geraniol-producing nudix hydrolases form *Rosa chinesis* (XP_02419729, XP_024185554). The sequenced identified by sequence similarity showed low identity along with minimal expression.

**
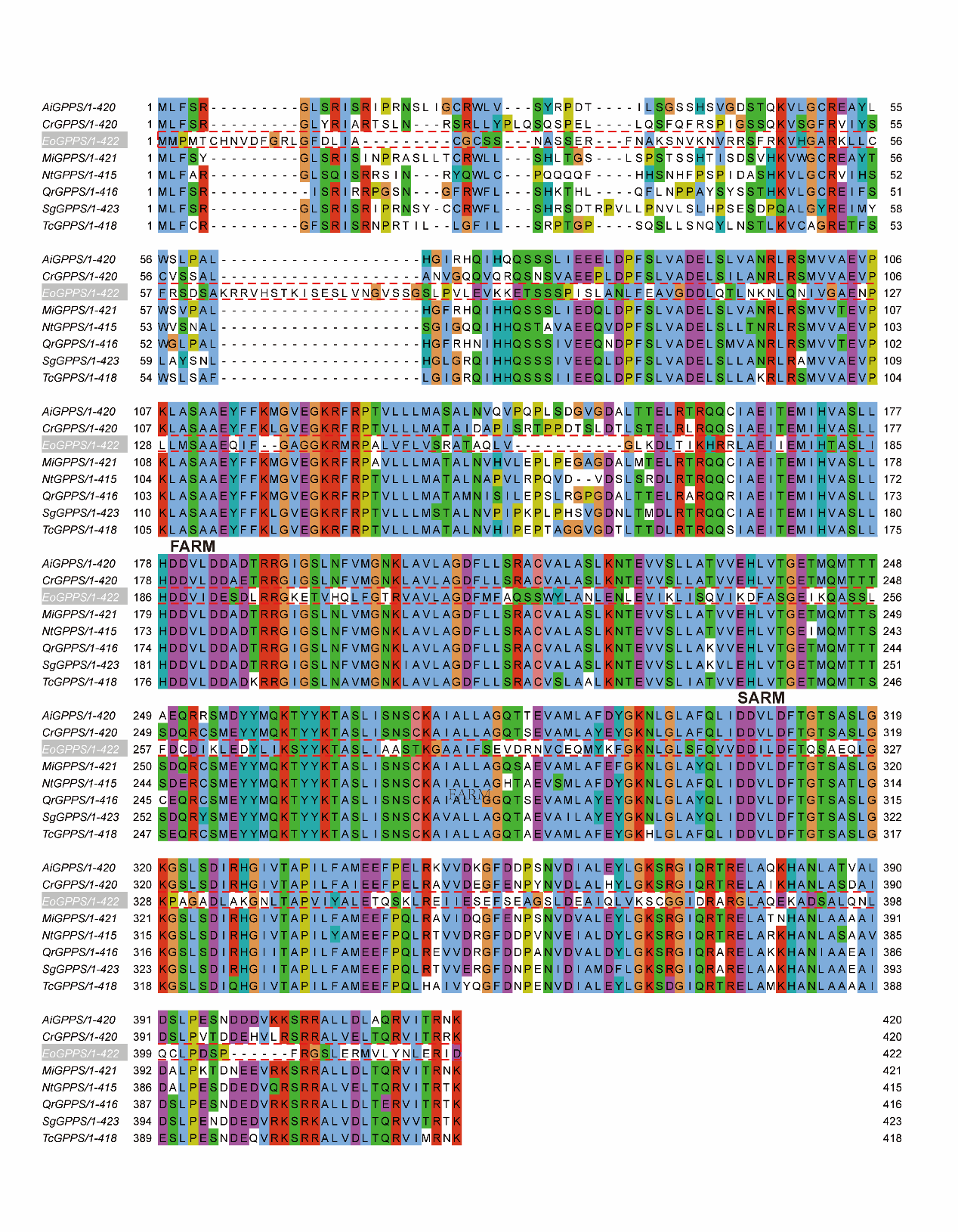
Figure S5. Sequence alignment for geranyl diphosphate synthase identified**

The sequence of geranyl diphosphate synthase identified in the current study (EoGPPS) is aligned to 7 other previously characterized geraniol synthases from *Theobroma cacao* (TcGPPS), *Siraitia grosvenorii* (SgGPPS), *Quercus robur* (QrGPPS), *Azadirachta indica* (AiGPPS), *Mangifera indica* (MiGPPS), *Nicotiana tabacum* (NtGPPS), *Catharanthus roseus* (CrGPPS). Conserved catalytic FARM and SARM motifs are marked.

**
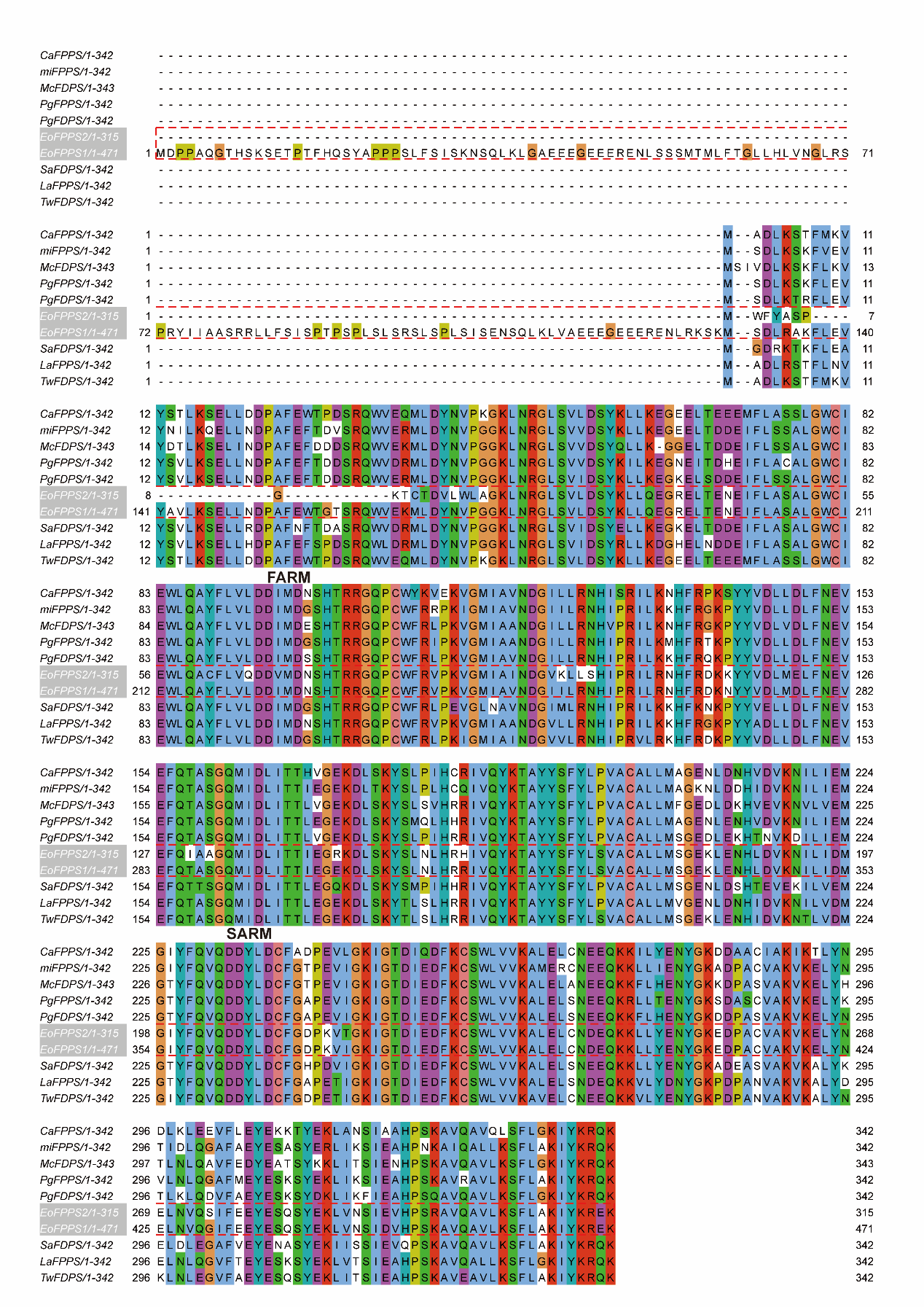
Figure S6. Sequence alignment for farnesyl diphosphate synthase identified**

The sequence of farnesyl diphosphate synthase identified in the current study (EoFPPS1, EoFPPS2) is aligned to 8 other previously characterized geraniol synthases from *Panax ginseng* (PgFDPS), *Mangifera indica* (MiFPPS), *Santalum album* (SaFDPS), *Lupinus albus* (LaFPPS), *Tripterygium wilfordii* (TwFDPS), *Matricaria chamomilla* var. recutita (McFDPS), and *Capsicum annuum* (CaFPPS). Conserved catalytic FARM and SARM motifs are marked.

**
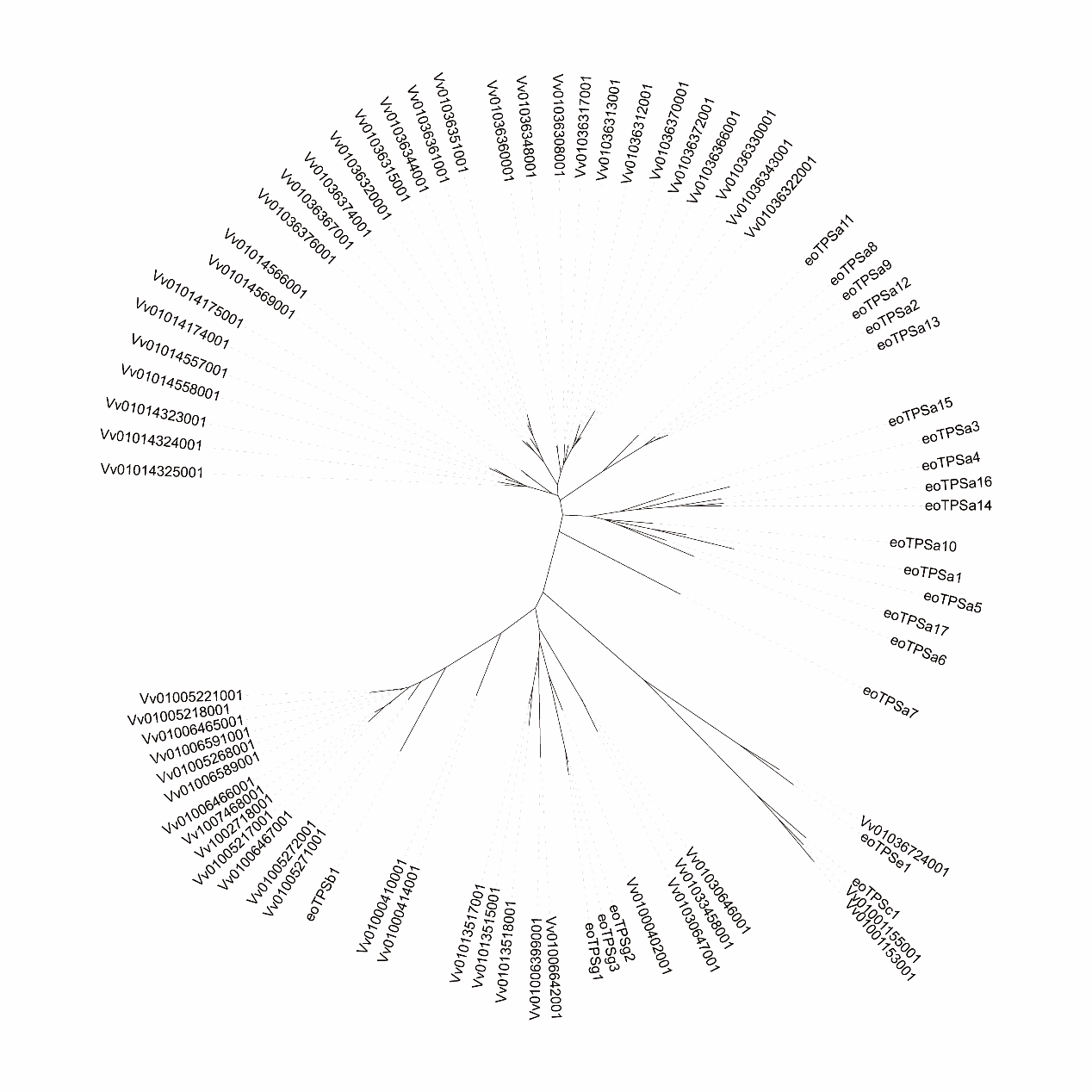
F****igure S7. Sequence clustering for terpene synthase identified**

Sequence clustering of terpene synthase identified in the current study along with terpene synthases from *Vitis Vinifera*. Terpene synthase from *Epiphyllum oxypetalum* were named according to subfamilies and expression level.

**
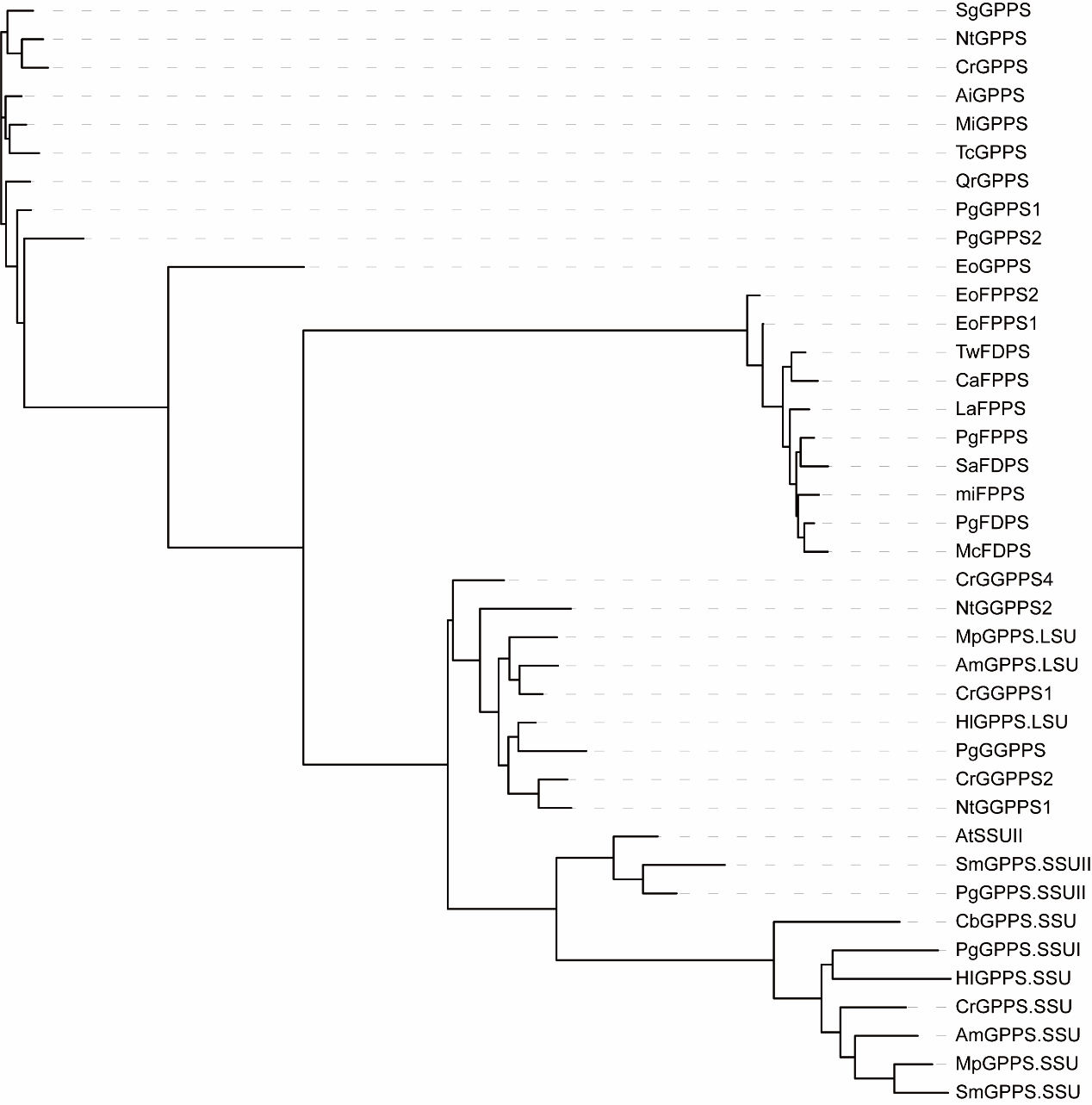
****Figure S8. Sequence clustering for isopentenyl diphosphate synthase identified**

Sequence clustering of eoGPPS, EoFPPS1 and EoFPPS2 with isopentyl diphosphates identified in other species. GPPS stands for homodimeric geranyl diosphate synthase; FPPS or FDPS stands for farnesyl diphosphate synthase; GGPPS stands for geranylgeranyl diphosphate synthase; GPPS.LSU stands for heterodimeric geranyl diphate synthase large subunit; GPPS.SSU stands for heterodimeric geranyl diphosphate synthase small subunit.
