# Supplementary material for: Floral scent emission of Epiphyllum oxypetalum: discovery of its cytosol-localized geraniol biosynthesis
Source: Hortic Res. 2025 Feb 11;12(5):uhaf039. doi: 10.1093/hr/uhaf039 (PMC11997432; doi:10.1093/hr/uhaf039)
Supplement: Web_Material_uhaf039 [file web_material_uhaf039.zip › hyperlinks-for-Sup-Movie.pdf]

Hyperlinks for Supplementary Movie S1

English version:

Time-lapse video of Epiphyllum oxypetalum blooming.mp4

https://disk.pku.edu.cn/link/AA52BC4947DC164EA78627FD704828A5AC

中文版:

昙花开放延时-中文.mp4

https://disk.pku.edu.cn/link/AA5D920FC90C1F46A0A5F4CBF2ECEADCDE
